# Physicians’ attention to patients’ communication cues can improve patient satisfaction with care and perception of physicians’ empathy

**DOI:** 10.1016/j.clinsp.2024.100377

**Published:** 2024-05-03

**Authors:** Carlos Frederico Confort Campos, Clarice Rosa Olivo, Milton de Arruda Martins, Patricia Zen Tempski

**Affiliations:** a The Centre for Medical and Health Sciences Education, University of Auckland, Auckland, New Zealand; b Center for Development of Medical Education, Universidade de São Paulo, Sao Paulo, SP, Brazil

**Keywords:** Health communication, Empathy, Patient satisfaction, Physician-patient relations, Nonverbal Communication

## Abstract

**Background:**

The pathway that links good communication skills and better health outcomes is still unclear. However, it is known that the way that physicians and patients communicate with each other has direct consequences on more “proximal outcomes”, such as perceptions of physician empathy and patient satisfaction. However, which specific communication skills lead to those patient outcomes is still unknown. In this study, the authors aimed to analyze which specific patient and physician communication skills are correlated to patients’ satisfaction with care and patient-perceived physician empathy.

**Methods:**

The authors classified and quantified verbal and nonverbal communication of second-year internal medicine residents and their patients through video recordings of their consultations. Patients also rated their satisfaction with care and the physician's empathy for them.

**Results:**

Using a linear regression model, the authors identified that patients’ and physicians’ expressions of disapproval, physicians’ disruptions, and patients’ use of content questions negatively correlated to patients’ satisfaction and patient-perceived physician empathy. Conversely, patient affective behaviors and the physician's provision of advice/suggestion were positively correlated to at least one of the patient-measured outcomes.

**Conclusion:**

Our findings point to the importance of physicians’ attentiveness to patients’ communication cues. Training physicians to interpret those cues could help develop more satisfactory and empathic therapeutic relationships.

## Highlights

• Patient-doctor communication relates to patient satisfaction and perceived empathy.
• Patients asking questions was linked to poor satisfaction and empathy perceptions.
• Doctor's use of negative communication skills related to worse rapport with patients.
• Satisfaction and empathy are positively linked to patient affective expression.
• Doctors providing advice/suggestions positively correlated to patient satisfaction.

## Introduction

The way that physicians and patients communicate with each other has important consequences. For the patient, good communication can lead to more satisfaction with the encounter, and perceptions that the physician empathizes with them.^1^ This helps develop a stronger relationship built on trust, which, in turn, will lead to more accurate diagnoses and greater compliance.^2^

Street et al. proposed a framework for how communication can affect health outcomes.^3^ In that framework, patient satisfaction and a sense of being “known” and understood (resultant of empathy as defined by Hojat)^4^ are considered “proximal outcomes”. Satisfaction and perceiving empathy are important measures that can positively impact “intermediate outcomes” such as patients’ sense of empowerment and the likelihood of complying with physicians’ recommendations.^3^^,^^5^^,^^6^ Conversely, poor physician-patient communication is associated with lower patient satisfaction,^7^ poorer adherence to treatment,^8^ more malpractice complaints and lawsuits,^9^ and worse objective and subjective (i.e., blood pressure and pain scales, respectively) health outcomes.^10^ In fact, good communication is considered a central skill for all physicians and other health professionals,^11^ performing two main functions: exchange of information and establishment of a relationship between patient and physician.^12^^,^^13^ A physician's empathy for their patient is seen by both physicians and patients as the basis of good communication.^14^

Different educational methods can help in improving physicians’ communication skills, perceptions of physician empathy, and patient satisfaction.^15^, ^16^, ^17^ Studies have reported different types of communication training targeted at physicians of different medical specialties, e.g., respiratory physicians and psychiatrists.^18^^,^^19^ More frequently, reports focus on a generic communication training workshop, such as emphasis on basic courtesy and summarization of findings in accessible language or patient engagement, education and problems in the patient-physician relationship.^17^^,^^20^ Occasionally, the focus is on broader topics such as breaking bad news or shared decision-making.^21^^,^^22^ However, to our knowledge, no studies have identified specific communication skills used by patients and physicians that relate to patient satisfaction with care and patient-perceived physician’ empathy.

Knowing which specific patient and physician communication skills lead to patient satisfaction with care and patient-perceived physicians’ empathy could inform the content for training future healthcare professionals. Therefore, the aim of this study is to analyze the relationship between a list of specified patient and physician communication skills, and patient's satisfaction with care, and patient-perceived physicians’ empathy. The authors asked the following research questions:

1) What is the relationship between scores of patient satisfaction with care and scores of a list of specified physician and patient communication skills?
2) What is the relationship between scores of patient perception of their physician's empathy and scores of a list of specified physician and patient communication skills?

## Methods

### Ethics

Our study was approved by the Ethics Committee of the School of Medicine of the University of São Paulo (Comissão de Ética para Análise de Projetos de Pesquisa ‒CAPPesq) and by the National Committee of Ethics in Research of the Ministry of Health of Brazil (Comissão Nacional de Ética em Pesquisa ‒ CONEP), protocol 2.825.441. All participating patients and residents read and signed the informed consent form.

### Study design

The authors undertook an explorative, cross-sectional survey-based study with quantitative analysis of data. This study is part of a larger mixed-methods project that studied the relationship between communication skills and physician-patient productive interactions.

### Context

The study was conducted in a preoperative risk assessment clinic in an outpatient ambulatory clinic from Hospital das Clínicas, a tertiary teaching hospital associated with the University of São Paulo in São Paulo, Brazil. The clinic is organized so patients are seen first by the internal medicine resident, who focuses on the assessment of the risk of clinical conditions, followed by an anesthetic consult, who will assess risks more directly related to the surgical and anesthetic procedures.

### Sample population

In Brazil, basic internal medicine training lasts two years after the conclusion of the undergraduate medical program. Our sample comprised seven second-year internal medicine residents doing a rotation at the preoperative risk assessment clinic ‒ mean age of 26.2 (SD = 1.2) years old, 42.9 % female ‒ and 10 patients enrolled in the clinic, whose characteristics are described in Table 1. The internal medicine training does not include any specific activity regarding healthcare communication or patient-physician relationship.

**Table 1:** Patients sample characteristics (*n* = 10).

Patients’ characteristics	*n* = 10
Age, mean (SD)	64.6 (10.3)
Gender, n (%)	
Female	6 (60.0)
Male	4 (40.0)
Educational level, n (%)	
Incomplete primary education	3 (30.0)
Complete primary education	1 (10.0)
Secondary education	4 (40.0)
Tertiary education	2 (20.0)
Postgraduate education	0 (0.0)
Surgical procedure by specialty, n (%)	
Ophthalmic surgery	3 (30.0)
Urology	3 (30.0)
Dermatologic surgery	2 (20.0)
Gynecology	1 (10.0)
Otorhinolaryngology	1 (10.0)
Clearance for procedure, n (%)	
Yes	7 (70.0)
No	3 (30.0)

SD, Standard Deviation.

The inclusion criteria for residents were being in the second year of the internal medicine program and allocated to the preoperative risk rotation clinic attachment during the study. There were no exclusion criteria for residents. All included patients were going to have a consultation with our sample of residents. The exclusion criteria for patients were significant cognitive, visual or auditive impairment.

Participation in the study was voluntary for members of both groups. No compensation was offered for participating.

### Sample approach

Data were collected between April and June 2019. All participating residents were invited at the beginning of the study days and agreed to participate in the study.

Patients who were going to have a consultation with our sample of residents were approached to rule out any exclusion factors. All eligible patients were invited to participate in the study.

### Sample size estimate

To our knowledge, no study has tried to quantify the correlation between specific patient and physician communication skills, and patient's satisfaction with care and patient-perceived physician's empathy using the same combination of tools the authors did. For that reason, we calculated the sample size of 10 videos based on a correlation coefficient of at least 0.80, using an alpha of 0.05 and a statistical power of 0.80.

### Measurement tools

The authors used the Brazilian version^23^ of the Jefferson Scale of Patient Perceptions of Physician Empathy (JSPPPE) to assess the patient's perception of their physician's empathy, translated by Bernardo et al. Authorization for the use of the scale was sought from the original authors, who provided us with the Brazilian version. An example item from the original scale is “My doctor listens carefully to me”. It comprises a five-item unidimensional tool using a seven-point Likert scale (with one being “strongly disagree” and seven being “strongly agree.”) that measures the physician's empathic engagement. Each item's response is summed, and the final score can range from 5 to 35. The internal consistency of the instrument is 0.98.^24^

To assess patient satisfaction with care, the authors used two tools: we asked patients to provide a Global Rating (GR) of their overall satisfaction with the consultation (a zero to 10 scale) and a Net Promoter Score (NPS) .^25^ For the latter, patients were asked to rate how likely they would be to recommend their physician to a friend or a colleague. The NPS were classified as promoters (9 and 10), passives (7 and 8), and detractors (six or less).

Created initially to measure user satisfaction, NPS has been used in the healthcare setting as a measure of user satisfaction with a healthcare service.^26^^,^^27^

### Data collection

After informed consent was obtained, physician-patient consultations were video recorded by a stand-alone camera (iPhone 4S, Apple, Cupertino, USA). Immediately following the consultation, patients were asked to complete the three measurements (JSPPPE, GR, and NPS). In addition, the authors collected sociodemographic data that included gender, age, educational level, and surgical procedure information (for which surgical procedure they were being assessed and whether they were cleared for that). From the resident group, the authors collected age and gender data. Our video sample is composed of 10 videos depicting the consultation between one pair of participating residents and a patient. Each patient appeared only in a single video. Four residents featured one video, and three residents featured two videos (each with a different patient).

### Assessment of communication skills during video-recorded physician-patient interactions

Verbal communication skills were analyzed using the Medical Communications Behaviour System.^28^ The system classifies every verbal utterance against 23 communication skills or behaviors, grouped into seven categories: 1) Physician content behaviors; 2) Physician affective behaviors; 3) Physician negative behaviors; 4) Patient content behavior; 5) Patient affective behavior; 6) Patient negative behavior and 7) Miscellaneous. Descriptions of the 23 communication behaviors can be found in Wolraich et al. (1986) .^28^ Nonverbal communication skills were analyzed using the following list of categories based on the behaviors described by Heintzman et al. .^29^ and Caris-Verhallen et al.^30^: 1) Forward leaning; 2) Affirmative head nodding; 3) Smiling; 4) Patient-directed eye gaze; 5) Affective touch and 6) Instrumental touch. The first three nonverbal skills were assessed separately for physicians and patients, and the last three were assessed for the dyad.

Two research team members (CC and CO) trained in the use of both verbal and nonverbal communication skills measurement tools. This involved independently evaluating training videos, and later discussing their evaluations with a third member of the research team (PT).

The two researchers then independently evaluated each video included in the study. The authors calculated Intraclass Correlation Coefficients (ICC) to quantify interrater reliability for each video and the whole group in both verbal and nonverbal skills assessments.

Each verbal utterance was classified against one of the 23 communication behaviors and timed (in seconds). The length of all occurrences of the same behavior was summed. This led to the total length of time each communication behavior appeared in each video. Then, the authors calculated the amount of time the evaluated subject (i.e., patient or physician) could be assessed for the specific behavior. It was done by subtracting the time the evaluated subject could not be assessed for the particular behavior (e.g., when the physician left the room or during the physical examination) from the total time of the consultation. Our final measurement, which the authors named net screen time, resulted from a division between each behavior's total length of time and the length of time the evaluated participant could be assessed for the specific behavior.

In a hypothetical example, a patient asked content questions twice with a length of one minute each and once for two seconds. Also, consider that the consultation lasted 10 minutes, but the patient was being examined for two minutes. That would account for a total of four minutes using the behavior and eight minutes that the patient could be assessed for the behavior “content question” (since for two minutes they were being examined and could not be assessed). Thus, for that consultation, the net screen time of content questions would be four divided by eight, resulting in 0.5 % or 50 %. This measurement could be interpreted as the patient spending 50 % of the consultation asking content questions.

The authors conducted similar calculations every time a nonverbal communication skill from our list was identified in the videos. The final net screen time value for every verbal and nonverbal communication skill was the average between the two researchers’ independent measurements.

### Data analysis

The authors presented all variables in descriptive statistics, with means and Standard Deviations (SD). The authors used a linear regression analysis to assess the association between the patient survey scores and the net screen time for each of the verbal and nonverbal communication skills. The authors used SPSS version 22.0 (IBM, Armonk, NY, USA) for data analysis and considered statistical significance at *p* < 0.05.

## Results

### Survey scores descriptive analysis

Patient perception of their physician's empathy measured by JSPPPE, patient satisfaction measured by GR, and by NPS showed a mean (SD) of 29.5 (7.9), 8.6 (3.1), and 8.2 (3.2), respectively (Table 2). NPS classification revealed that our sample had 2 (20.0 %) detractors, 3 (30.0 %) passives, and 5 (50.0 %) promoters.

**Table 2:** Patients’ assessment of their Physician's Empathy (JSPPPE) and their satisfaction with care (GR and NPS).

Patient from video	JSPPPE^a^	GR^b^	NPS^b^
1	8	0	0
2	31	8	6
3	32	10	10
4	34	10	10
5	29	10	7
6	30	8	8
7	35	10	10
8	29	10	8
9	35	10	10
10	32	10	10

JSPPPE, Jefferson Scale of Patient Perceptions of Physician Empathy; GR, Global Rating; NPS, Net Promoter Score.

a Ranges from 5 to 35.

b Ranges from zero to 10.

### Communication skills analysis

The assessment of communication skills of the consultation videos included in the study conducted by two independent researchers demonstrated excellent interrater reliability.^31^ The ICC for verbal and nonverbal communication assessment of the set of 10 videos resulted in ICC of 0.980 and 0.959, respectively (Supplementary Table 1).

The final net screen time for all verbal communication skills in each video is shown in Supplementary Table 2. The corresponding net screen times for the nonverbal skill are presented in Supplementary Table 3.

The linear regression analysis revealed that different communication skills correlated either positively (Table 3) or negatively (Table 4) with the three survey measures (JSPPPE, GR and NPS). JSPPPE, GR, and NPS showed a significant negative association with physician disapproval, physician disruptions, physician negative behaviors, patient content questions, patient disapproval and unclassified communication skills. Conversely, JSPPPE, GR and NPS presented with a positive significant association with patient affective behaviors. NPS was also positively associated with the use of advice/suggestions. There was no association between any nonverbal communication skills and the three measures. The linear regression coefficients for all communication skills can be found in Supplementary Tables 4 and 5.

**Table 3:** Linear regression model for the communication skills positively associated with three survey measures (JSPPPE, GR and NPS).

		JSPPPE			GR			NPS		
Related to Skill		R^2^	β	p	R^2^	β	p	R^2^	β	p
Physician	Advice/suggestion	‒	‒	‒	‒	‒	‒	0.428	1.276	0.040
Patient	Patient affective behaviors	0.447	0.006	0.035	0.597	0.003	0.009	0.674	0.003	0.004

JSPPPE, Jefferson Scale of Patient Perceptions of Physician Empathy; GR, Global Rating; NPS, Net Promoter Score; β, Standardized Coefficient; R^2^, Coefficient of Determination.

**Table 4:** Linear regression model for the communication skills negatively associated with three survey measures (JSPPPE, GR and NPS).

		JSPPPE			GR			NPS		
Related to Skill		R^2^	β	p	R^2^	β	p	R^2^	β	p
Physician	Disapproval	0.824	−20.046	<0.001	0.807	−7.893	<0.001	0.630	−6.995	0.006
	Disruptions	0.917	−4.606	<0.001	0.960	−1.875	<0.001	0.779	−1.694	0.001
	Physician negative behaviors	0.919	−3.835	<0.001	0.950	−1.551	<0.001	0.764	−1.395	0.001
Patient	Content questions	0.871	−3.453	<0.001	0.821	−1.334	<0.001	0.843	−1.356	<0.001
	Disapproval	0.587	−9.507	0.010	0.684	−4.085	0.003	0.650	−3.992	0.005
Both	Unclassified	0.727	−1.966	0.002	0.747	−0.793	0.001	0.731	−0.786	0.002

JSPPPE, Jefferson Scale of Patient Perceptions of Physician Empathy; GR, Global Rating; NPS, Net Promoter Score; β, Standardized Coefficient; R^2^, Coefficient of Determination.

## Discussion

The objective of this study was to analyze the relationship between a specific list of patient and physician communication skills, and patient satisfaction with care and patients-perceived physician empathy. The authors found that patient and physician negative behaviors, such as disapproval or disruptions, negatively correlated with patient satisfaction and perceived empathy. The use of content questions by patients also showed a similar negative correlation. Conversely, patient affective behaviors were positively correlated to the patient-measured outcomes. Finally, physicians’ behaviors of giving advice or suggestions positively correlated with patient satisfaction. The authors found no correlation between any of the nonverbal communication behaviors and patient satisfaction or perceived empathy of their physician.

The negative correlation of patient satisfaction with both patient and physician disapproval and physician disruptions is not unexpected. Wolraich et al. defines disapproval (either by the physician or the patient) as “rejection or criticism” towards the other party, “sarcasm, and ignoring” their feelings.^28^ A review from Williams et al. .^32^ reported studies pointing in the same direction. Although published in 1998, it is still one of the most significant reviews on the topic of patient-physician communication. The use of a negative tone by either physicians or patients or physicians expressing disagreement or anger was associated with patient dissatisfaction. Our finding of a similar correlation with patient perception of empathy is also unsurprising. However, the literature on the link between specific communication skills and empathy is very scarce. A study reported by Torain et al. found that for ambulatory patients in a large academic center in the United States, their physician's use of ‘hurried communication’ correlated negatively with patient perception of their provider's empathy,^33^ resonating with our findings.

Wolraich et al. defines content questions as “questions that seek information, advice, or clarification from the medical team” .^28^ Our finding that patients asking those questions to their providers presented with a negative correlation with their satisfaction and perception of their physician's empathy requires more understanding, especially in light of our additional finding of a positive correlation between physician advice/suggestion and patient satisfaction. Studies often report the link between patient and physician communication skills related to the exchange of information and patient satisfaction and their physicians’ perceived empathy.^34^ However, these studies tend to focus on the aspect of the physician giving information. In a systematic review, McMillan et al. .^35^ reported that the satisfaction of patients with chronic conditions is strongly related to the amount of information given by the physician, in a patient-centered approach. Torain et al. used questionnaires to gather patients’ perceptions of their care. They found that physicians who spent more time explaining information such as results or medication were seen as more empathetic.^33^ Likewise, in a study performed with cancer patients in an important cancer care center in the United States, Sanders et al. found that they see a physicians as empathetic if they take the time to explain ‘everything’ to them.^36^

Fewer studies have focused on the patient “side” of the interaction. To our knowledge, the seminal study that found the negative association between patients who asked more questions and less satisfied with the received was conducted by Roter^37^ in a primary care setting. Later Venetis et al. ,^38^ in a study conducted with breast cancer patients, found something similar. They go on to report that very few studies have explored that association in different clinical settings. One might think that in asking more questions, patients would receive more information from their physicians and thus be more satisfied.^39^ However, the authors hypothesize that the increased number of questions could also be seen as an expression of dissatisfaction if patients think they have been offered too little information. It could also point to the presence of some level of mistrust in the physician, requiring them to ask more questions to reassure themselves that their physician is focused on their care and knows what they are doing.

In our study, patients’ expression of affective behaviors positively correlated to their satisfaction with care and perception of their physician's empathy. Studies on the association between the expression of affective behaviors and patient-measured satisfaction and empathy are limited. Again, most papers focus on the physician's side of effective communication.^40^ One review from Williams et al. reported that physicians’ friendliness or use of social conversation was related to patient satisfaction.^32^ Torain et al. also identified that when a provider's communication style is perceived as compassionate and respectful, patients perception of their empathy is improved.^33^ The authors found one study by Haskard et al. on the effect of the patient expressing affective behaviors, in which the authors found a correlation between patients expressing more “pleasant” affects and their satisfaction with primary care nurses' interpersonal care and competence.^41^

### Limitations

Our study has some limitations. Communication is a complex construct, and the authors reduced it to a series of measurable skills, which may not fully represent the whole. However, as in any type of scientific methodology, the authors had to take a focused approach. The authors decided to use a quantitative method to be able to measure something as comprehensive as communication. This brought us closer to the methodology used in other important literature. There is also a possibility of bias in our rating of communication skills; however, the authors mitigated this through rater training. Moreover, our sample comprises a small number of patients and physicians and their video-recorded interactions. Although the literature^42^ and our sample size calculation support our study, a study with a larger sample would be able to identify other associations. Finally, the study was conducted in a single institution, which could diminish its generalizability.

### Future research

Patient satisfaction with care and their perceptions of physician empathy are important proximal outcomes that can positively influence intermediate and final health outcomes. Our study furthers the discussion on identifying specific patient and physician communication behaviors that correlate with these two proximal outcomes.

Future research could further explore the connection between those communication behaviors and patient-centered outcomes. Our study suggests that patients react in a way that reflects their satisfaction (or dissatisfaction) with the medical encounter – and their reaction also reflects the empathy they attribute to their provider. Studies should focus on how to train physicians to interpret their patients’ communication and reactions to them, as these can signal positive or negative patient satisfaction or patients’ perceptions of lack of empathy.

Physicians should also be trained to be mindful of somewhat obvious behaviors that negatively affect their rapport with patients, such as disruptions or even expressions of disapproval of their patients.

Finally, the link between patients asking questions and negative evaluation of the consultation and the provider certainly needs further investigation. Although our finding is supported by literature, the connection is yet to be understood. The authors hypothesize that patient questions may, in some circumstances, reflect dissatisfaction with the explanation or information provided by the physician.

## CRediT authorship contribution statement

**Carlos Frederico Confort Campos:** Conceptualization, Data curation, Formal analysis, Investigation, Methodology, Project administration, Resources, Validation, Visualization, Writing – original draft, Writing – review & editing. **Clarice Rosa Olivo:** Formal analysis, Investigation, Validation, Writing – review & editing. **Milton de Arruda Martins:** Conceptualization, Methodology, Resources, Supervision, Writing – review & editing. **Patricia Zen Tempski:** Conceptualization, Methodology, Project administration, Resources, Supervision, Validation, Writing – review & editing.

## Conflicts of interest

The authors declare no conflicts of interest.

## Appendix Supplementary materials

Image, application 1
