# Supplementary material for: Physicians’ attention to patients’ communication cues can improve patient satisfaction with care and perception of physicians’ empathy
Source: Clinics (Sao Paulo). 2024 May 3;79:100377. doi: 10.1016/j.clinsp.2024.100377 (PMC11087704; doi:10.1016/j.clinsp.2024.100377)
Supplement: Supplementary file 1 [file mmc1.docx]

**CLINICS-D-23-00635_Supplementary Material**

**Supplementary Table 1** Intraclass correlation coefficients calculated by the independent assessors of communication skills by videos and types of skills.

| **Video** | **Verbal skills** | | **Non-verbal skills** | |
| --- | --- | --- | --- | --- |
|  | **ICC** | **p** | **ICC** | **p** |
| 1 | 0.978 | <0.001 | 0.927 | <0.001 |
| 2 | 0.983 | <0.001 | 0.956 | <0.001 |
| 3 | 0.998 | <0.001 | 0.990 | <0.001 |
| 4 | 0.998 | <0.001 | 0.943 | <0.001 |
| 5 | 0.980 | <0.001 | 0.923 | <0.001 |
| 6 | 0.978 | <0.001 | 0.903 | <0.001 |
| 7 | 0.983 | <0.001 | 0.932 | <0.001 |
| 8 | 0.941 | <0.001 | 0.994 | <0.001 |
| 9 | 0.974 | <0.001 | 0.990 | <0.001 |
| 10 | 0.989 | <0.001 | 0.984 | <0.001 |
| Whole set | 0.980 | <0.001 | 0.959 | <0.001 |

ICC, Intraclass correlation Coefficient.

**Supplementary Table 2** Final net screen time for all verbal communication skills and categories.

| **Skills/Category** | **Videos** | | | | | | | | | |
| --- | --- | --- | --- | --- | --- | --- | --- | --- | --- | --- |
|  | **1** | **2** | **3** | **4** | **5** | **6** | **7** | **8** | **9** | **10** |
| Physician Content Behaviors | 24.4% | 28.3% | 28.9% | 29.6% | 28.8% | 21.3% | 29.8% | 17.2% | 21.8% | 21.6% |
| History/background probes | 4.6% | 14.0% | 10.8% | 12.3% | 2.6% | 7.9% | 10.8% | 11.7% | 8.6% | 9.9% |
| Checks for understanding | 0.0% | 0.0% | 0.0% | 0.0% | 1.4% | 0.2% | 0.0% | 0.0% | 0.0% | 0.0% |
| Information | 19.1% | 11.1% | 13.6% | 11.0% | 21.8% | 8.4% | 14.3% | 5.3% | 9.2% | 9.9% |
| Advice/suggestion | 0.0% | 1.0% | 3.7% | 3.5% | 2.1% | 4.0% | 3.8% | 0.0% | 3.6% | 1.3% |
| Restatement | 0.7% | 2.2% | 0.8% | 2.7% | 0.6% | 0.8% | 0.9% | 0.1% | 0.4% | 0.6% |
| Clarification | 0.0% | 0.0% | 0.0% | 0.0% | 0.2% | 0.0% | 0.0% | 0.0% | 0.0% | 0.0% |
| Physician Affective Behaviors | 0.8% | 4.4% | 0.9% | 3.4% | 2.7% | 3.2% | 1.8% | 2.4% | 2.0% | 3.9% |
| Emotional probes | 0.0% | 0.0% | 0.0% | 0.0% | 0.0% | 0.0% | 0.0% | 0.0% | 0.0% | 0.0% |
| Reassurance/support | 0.0% | 0.0% | 0.1% | 0.2% | 1.9% | 1.4% | 0.0% | 0.0% | 0.0% | 0.2% |
| Reflection of feelings | 0.0% | 0.0% | 0.0% | 0.0% | 0.1% | 0.0% | 0.0% | 0.0% | 0.0% | 0.0% |
| Encourages | 0.8% | 4.4% | 0.8% | 3.2% | 0.7% | 1.8% | 1.8% | 2.4% | 2.0% | 3.7% |
| Physician Negative Behaviors | 6.3% | 0.0% | 0.5% | 0.0% | 0.0% | 1.0% | 0.0% | 0.0% | 0.0% | 0.1% |
| Disapproval | 1.1% | 0.0% | 0.4% | 0.0% | 0.0% | 0.0% | 0.0% | 0.0% | 0.0% | 0.0% |
| Disruptions | 5.2% | 0.0% | 0.0% | 0.0% | 0.0% | 1.0% | 0.0% | 0.0% | 0.0% | 0.0% |
| Jargon | 0.0% | 0.0% | 0.0% | 0.0% | 0.0% | 0.0% | 0.0% | 0.0% | 0.0% | 0.1% |
| Patient Content Behaviors | 36.6% | 43.6% | 40.2% | 32.3% | 44.4% | 27.5% | 18.8% | 32.9% | 36.6% | 49.0% |
| Content questions | 7.2% | 1.1% | 0.2% | 0.3% | 2.8% | 0.8% | 1.4% | 0.6% | 0.4% | 0.5% |
| Content remarks | 27.9% | 40.9% | 39.8% | 31.9% | 37.9% | 25.4% | 17.3% | 32.4% | 35.3% | 48.2% |
| Checks for understanding | 1.5% | 1.5% | 0.2% | 0.2% | 3.8% | 1.2% | 0.0% | 0.0% | 0.9% | 0.3% |
| Patient Affective Behaviors | 19.7% | 16.8% | 15.6% | 14.4% | 11.5% | 10.1% | 9.3% | 5.7% | 4.6% | 1.2% |
| Encourages | 2.8% | 1.2% | 0.9% | 1.2% | 0.5% | 0.3% | 3.6% | 1.1% | 3.3% | 0.7% |
| Emotional expressions | 0.1% | 0.0% | 0.3% | 1.7% | 0.9% | 0.5% | 0.0% | 0.0% | 0.1% | 0.5% |
| Patient Negative Behaviors | 1.8% | 1.1% | 0.8% | 0.0% | 0.0% | 0.0% | 16.6% | 0.0% | 0.0% | 0.0% |
| Disapproval | 1.8% | 1.1% | 0.8% | 0.0% | 0.0% | 0.0% | 0.0% | 0.0% | 0.0% | 0.0% |
| Disruptions | 0.0% | 0.0% | 0.0% | 0.0% | 0.0% | 0.0% | 16.6% | 0.0% | 0.0% | 0.0% |
| Miscellaneous |  |  |  |  |  |  |  |  |  |  |
| Social amenities | 4.0% | 9.1% | 0.6% | 3.8% | 0.4% | 18.8% | 1.4% | 6.4% | 1.4% | 7.1% |
| Silence | 14.4% | 6.6% | 18.0% | 20.6% | 23.0% | 20.0% | 29.1% | 37.8% | 31.9% | 8.7% |
| Unclassifiable | 13.2% | 6.3% | 5.6% | 5.2% | 4.4% | 2.3% | 3.8% | 3.1% | 1.3% | 1.7% |

**Supplementary Table 3** Final calculated net screen time for all non-verbal communication skills.

| **Skills** | **Videos** | | | | | | | | | |
| --- | --- | --- | --- | --- | --- | --- | --- | --- | --- | --- |
|  | **1** | **2** | **3** | **4** | **5** | **6** | **7** | **8** | **9** | **10** |
| Physician assessed skill |  |  |  |  |  |  |  |  |  |  |
| Forward leaning | 0.0% | 0.0% | 2.9% | 0.0% | 14.5% | 0.0% | 0.0% | 1.7% | 0.0% | 0.0% |
| Affirmative head nodding | 4.4% | 1.1% | 4.7% | 7.6% | 10.2% | 4.6% | 5.4% | 14.7% | 12.3% | 10.2% |
| Smiling | 0.7% | 4.4% | 1.9% | 5.8% | 0.1% | 6.1% | 3.0% | 1.5% | 1.7% | 6.8% |
| Physician assessed skill |  |  |  |  |  |  |  |  |  |  |
| Forward leaning | 0.8% | 6.0% | 0.1% | 1.8% | 0.0% | 8.1% | 16.7% | 0.0% | 1.4% | 0.0% |
| Affirmative head nodding | 1.8% | 6.0% | 4.8% | 4.7% | 7.1% | 4.0% | 2.6% | 1.0% | 4.1% | 0.2% |
| Smiling | 2.1% | 1.3% | 2.9% | 7.7% | 0.1% | 11.2% | 11.4% | 1.0% | 0.8% | 4.8% |
| Commonly assessed skills |  |  |  |  |  |  |  |  |  |  |
| Patient-directed eye gaze | 23.8% | 28.9% | 34.8% | 19.3% | 39.2% | 7.5% | 17.3% | 34.1% | 28.5% | 17.9% |
| Affective touch | 0.0% | 0.4% | 0.3% | 0.0% | 0.1% | 0.0% | 0.5% | 0.7% | 0.1% | 0.0% |
| Instrumental touch | 0.0% | 0.0% | 0.0% | 0.0% | 0.0% | 0.0% | 0.0% | 7.7% | 4.4% | 0.0% |

**Supplementary Table 4** Linear regression analysis for patient-perceived doctor’s empathy (measured by JSPPPE), and patient satisfaction with care (measured by GR and NPS) among verbal communication skills.

| **Communication skill** | **JSPPPE** | | | **GR** | | | **NPS** | | |
| --- | --- | --- | --- | --- | --- | --- | --- | --- | --- |
|  | **R^2^** | **β** | **p** | **R^2^** | **β** | **p** | **R^2^** | **β** | **p** |
| Physician content behaviors | 0.022 | 0.263 | 0.679 | 0.005 | 0.048 | 0.851 | 0.004 | 0.041 | 0.871 |
| History/background probes | 0.282 | 1.190 | 0.114 | 0.167 | 0.364 | 0.241 | 0.219 | 0.418 | 0.173 |
| Checks for understanding | <0.001 | -0.332 | 0.959 | 0.021 | 1.039 | 0.687 | 0.010 | -0.707 | 0.785 |
| Information | 0.222 | -0.742 | 0.169 | 0.165 | -0.254 | 0.244 | 0.237 | -0.306 | 0.154 |
| Advice/suggestion | 0.388 | 3.046 | 0.054 | 0.238 | 0.948 | 0.153 | 0.428 | 1.276 | 0.040^a^ |
| Restatement | 0.045 | 2.024 | 0.558 | 0.003 | 0.207 | 0.882 | 0.005 | 0.264 | 0.850 |
| Clarification | <0.001 | -2.525 | 0.951 | 0.025 | 7.071 | 0.665 | 0.010 | -4.545 | 0.782 |
| Physician affective behaviors | 0.189 | 2.844 | 0.209 | 0.138 | 0.967 | 0.291 | 0.066 | 0.671 | 0.474 |
| Emotional probes | N/A | N/A | N/A | N/A | N/A | N/A | N/A | N/A | N/A |
| Reassurance/support | 0.001 | 0.305 | 0.942 | 0.017 | 0.589 | 0.721 | 0.001 | -0.123 | 0.941 |
| Reflection of feelings | <0.001 | -5.051 | 0.951 | 0.025 | 14.141 | 0.665 | 0.010 | -9.091 | 0.782 |
| Encourages/acknowledge | 0.156 | 2.434 | 0.258 | 0.076 | 0.673 | 0.442 | 0.067 | 0.637 | 0.469 |
| Physician negative behaviors | 0.919 | -3.835 | <0.001^a^ | 0.950 | -1.551 | <0.001^a^ | 0.764 | -1.395 | 0.001^a^ |
| Disapproval | 0.824 | -20.046 | <0.001^a^ | 0.807 | -7.893 | <0.001^a^ | 0.630 | -6.995 | 0.006^a^ |
| Disruptions | 0.917 | -4.606 | <0.001^a^ | 0.960 | -1.875 | <0.001^a^ | 0.779 | -1.694 | 0.001^a^ |
| Jargon | 0.028 | 62.500 | 0.644 | 0.055 | 35.000 | 0.513 | 0.124 | 52.500 | 0.318 |
| Patient content behaviors | 0.014 | -0.105 | 0.746 | <0.001 | -0.002 | 0.985 | 0.019 | -0.049 | 0.704 |
| Content questions | 0.871 | -3.453 | <0.001^a^ | 0.821 | -1.334 | <0.001^a^ | 0.843 | -1.356 | <0.001^a^ |
| Content remarks | 0.022 | 0.133 | 0.683 | 0.059 | 0.087 | 0.499 | 0.021 | 0.052 | 0.690 |
| Checks for understanding | 0.094 | -2.051 | 0.389 | 0.052 | 0.604 | 0.528 | 0.212 | -1.228 | 0.181 |
| Patient affective behaviors | 0.447 | 0.006 | 0.035^a^ | 0.597 | 0.003 | 0.009^a^ | 0.674 | 0.003 | 0.004^a^ |
| Encourages | 0.017 | -0.854 | 0.721 | 0.069 | -0.687 | 0.464 | 0.015 | -0.321 | 0.736 |
| Emotional expressions | 0.039 | 2.899 | 0.586 | 0.055 | 1.374 | 0.514 | 0.059 | 1.432 | 0.497 |
| Patient negative behaviors | 0.024 | 0.237 | 0.668 | 0.003 | 0.035 | 0.874 | 0.020 | 0.085 | 0.700 |
| Disapproval | 0.587 | -9.506 | 0.010^a^ | 0.684 | -4.085 | 0.003^a^ | 0.650 | -3.992 | 0.005^a^ |
| Disruptions | 0.060 | 0.368 | 0.495 | 0.025 | 0.094 | 0.665 | 0.055 | 0.140 | 0.514 |
| Miscellaneous |  |  |  |  |  |  |  |  |  |
| Social amenities | 0.001 | -0.050 | 0.923 | 0.018 | -0.076 | 0.710 | 0.011 | -0.059 | 0.774 |
| Silence | 0.062 | 0.199 | 0.487 | 0.348 | 0.110 | 0.324 | 0.095 | 0.098 | 0.386 |
| Unclassifiable | 0.727 | -1.966 | 0.002^a^ | 0.747 | -0.793 | 0.001^a^ | 0.731 | -0.786 | 0.002^a^ |

JSPPPE, Jefferson Scale of Patient Perceptions of Physician Empathy; GR, Global Rating; NPS, Net Promoter Score; β, Standardized Coefficient; R^2^, Coefficient of Determination; N/A, Not Applicable.

^a^ p < 0.05.

**Supplementary Table 5** Linear regression analysis for patient-perceived doctor’s empathy (measured by JSPPPE), and patient satisfaction with care (measured by GR and NPS) among non-verbal communication skills.

| **Communication skill** | **JSPPPE** | | | **GR** | | | **NPS** | | |
| --- | --- | --- | --- | --- | --- | --- | --- | --- | --- |
|  | **R^2^** | **β** | **p** | **R^2^** | **β** | **p** | **R^2^** | **β** | **p** |
| Physician assessed skill |  |  |  |  |  |  |  |  |  |
| Forward leaning | <0.001 | -0.004 | 0.995 | 0.044 | 0.144 | 0.562 | 0.003 | -0.036 | 0.885 |
| Affirmative head nodding | 0.048 | 0.410 | 0.542 | 0.174 | 0.310 | 0.230 | 0.114 | 0.252 | 0.340 |
| Smiling | 0.159 | 1.309 | 0.253 | 0.064 | 0.330 | 0.481 | 0.162 | 0.526 | 0.250 |
| Patient assessed skill |  |  |  |  |  |  |  |  |  |
| Forward leaning | 0.073 | 0.392 | 0.450 | 0.007 | 0.048 | 0.819 | 0.027 | 0.095 | 0.652 |
| Affirmative head nodding | 0.063 | 0.904 | 0.483 | 0.045 | 0.301 | 0.558 | 0.003 | 0.073 | 0.890 |
| Smiling | 0.075 | 0.502 | 0.444 | 0.015 | 0.090 | 0.735 | 0.110 | 0.242 | 0.349 |
| Commonly assessed skills |  |  |  |  |  |  |  |  |  |
| Patient-directed eye gaze | 0.002 | -0.036 | 0.901 | 0.022 | 0.048 | 0.683 | 0.009 | -0.031 | 0.790 |
| Affective touch | 0.066 | 7.931 | 0.475 | 0.097 | 3.893 | 0.381 | 0.027 | 2.012 | 0.653 |
| Instrumental touch | 0.012 | 0.323 | 0.764 | 0.051 | 0.265 | 0.531 | 0.018 | 0.158 | 0.712 |

JSPPPE, Jefferson Scale of Patient Perceptions of Physician Empathy; GR, Global Rating; NPS, Net Promoter Score; β, Standardized Coefficient; R^2^, Coefficient of Determination.
